# Construction of a comparative genetic map in faba bean (*Vicia faba *L.); conservation of genome structure with *Lens culinaris*

**DOI:** 10.1186/1471-2164-9-380

**Published:** 2008-08-09

**Authors:** Simon R Ellwood, Huyen TT Phan, Megan Jordan, James Hane, Anna M Torres, Carmen M Avila, Serafín Cruz-Izquierdo, Richard P Oliver

**Affiliations:** 1 Australian Centre for Necrotrophic Fungal Pathogens, State Agricultural Biotechnology Centre, Health Sciences, Murdoch University 6150, Western Australia; 2 IFAPA, Centro Alameda del Obispo, Area de Mejora y Biotecnología, Apdo. 3092, E-14080 Córdoba, Spain

## Abstract

**Background:**

The development of genetic markers is complex and costly in species with little pre-existing genomic information. Faba bean possesses one of the largest and least studied genomes among cultivated crop plants and no gene-based genetic maps exist. Gene-based orthologous markers allow chromosomal regions and levels of synteny to be characterised between species, reveal phylogenetic relationships and chromosomal evolution, and enable targeted identification of markers for crop breeding. In this study orthologous codominant cross-species markers have been deployed to produce the first exclusively gene-based genetic linkage map of faba bean (*Vicia faba*), using an F_6 _population developed from a cross between the lines Vf6 (*equina *type) and Vf27 (*paucijuga *type).

**Results:**

Of 796 intron-targeted amplified polymorphic (ITAP) markers screened, 151 markers could be used to construct a comparative genetic map. Linkage analysis revealed seven major and five small linkage groups (LGs), one pair and 12 unlinked markers. Each LG was comprised of three to 30 markers and varied in length from 23.6 cM to 324.8 cM. The map spanned a total length of 1685.8 cM. A simple and direct macrosyntenic relationship between faba bean and *Medicago truncatula *was evident, while faba bean and lentil shared a common rearrangement relative to *M. truncatula*. One hundred and four of the 127 mapped markers in the 12 LGs, which were previously assigned to *M. truncatula *genetic and physical maps, were found in regions syntenic between the faba bean and *M. truncatula *genomes. However chromosomal rearrangements were observed that could explain the difference in chromosome numbers between these three legume species. These rearrangements suggested high conservation of *M. truncatula *chromosomes 1, 5 and 8; moderate conservation of chromosomes 2, 3, 4 and 7 and no conservation with *M. truncatula *chromosome 6. Multiple PCR amplicons and comparative mapping were suggestive of small-scale duplication events in faba bean. This study also provides a preliminary indication for finer scale macrosynteny between *M. truncatula*, lentil and faba bean. Markers originally designed from genes on the same *M. truncatula *BACs were found to be grouped together in corresponding syntenic areas in lentil and faba bean.

**Conclusion:**

Despite the large size of the faba bean genome, comparative mapping did not reveal evidence for polyploidisation, segmental duplication, or significant rearrangements compared to *M. truncatula*, although a bias in the use of single locus markers may have limited the detection of duplications. Non-coding repetitive DNA or transposable element content provides a possible explanation for the difference in genome sizes. Similar patterns of rearrangements in faba bean and lentil compared to *M. truncatula *support phylogenetic studies dividing these species into the tribes Viceae and Trifoliae. However, substantial macrosynteny was apparent between faba bean and *M. truncatula*, with the exception of chromosome 6 where no orthologous markers were found, confirming previous investigations suggesting chromosome 6 is atypical. The composite map, anchored with orthologous markers mapped in *M. truncatula*, provides a central reference map for future use of genomic and genetic information in faba bean genetic analysis and breeding.

## Background

Faba bean (*Vicia faba *L.) is currently the third most important cool-season food legume in the world. Faba bean provides an important source of dietary protein in human diet, edible oil and animal feeds. Like other grain legumes, faba bean contributes to sustainable agriculture in the management of soil fertility and plays an essential role in crop rotation. Faba bean is a diploid with 2n = 2x = 12 chromosomes [1,2], is partially cross-pollinated ranging from 4 to 84% [3], and possesses one of the largest genomes among crop legumes (~13000 Mb). The development of saturated linkage maps as well as the identification and map-based isolation of important qualitative traits or quantitative trait loci is therefore complex and expensive.

A saturated genetic linkage map provides an invaluable tool in plant genetic studies and practical breeding. One of the first genetic linkage maps of faba bean was constructed by Van de Ven *et al*. [4] with only 17 markers; followed by successively more detailed genetic maps by Torres *et al*. [5] with 51 markers, Satovic *et al*. [6] with 157 markers, Vaz Patto *et al*. [7] with 116 markers, Roman *et al*. [8] with 121 markers, Roman *et al*. [9] with 192 markers and Avila *et al*. [10] with 103 markers. To date, faba bean genetic maps have been restricted to morphological, isozyme, RFLP, RAPD, a few seed protein genes and four SSR markers. These markers have been limited either in number, transferability or in their ability to provide syntenic information with other legume species.

The recent generation of abundant genomic and genetic resources focussed around the model species *Medicago truncatula *and *Lotus Japonicus *has opened up abundant opportunities for creating gene-based molecular markers that are ideal for genetic mapping in general and comparative mapping in particular [11-16]. In this approach, oligonucleotide primers were designed from sequences of conserved regions in gene exons that flank polymorphic regions such as introns or microsatellites. This PCR-based, codominant marker system has remarkably increased the efficiency of transferring genetic information across species. Examples include the comparison of *M. truncatula *with alfalfa, pea, chickpea, soybean, mung bean, lentil and lupins [12-18].

Comparative genomic studies can expose and confirm phylogenetic relationships among species and determine patterns of chromosomal evolution and syntenic relationships. More importantly, comprehensive comparative genomics can facilitate back-and-forth use of genomic resources between different legumes species, and help to reduce cost and increase efficiency in genetic research as well as crop breeding. The use of conserved genome structure to assist in transferring knowledge among related plant species is well established in grasses [19,20] where synteny greatly assists in gene identification among related species.

In this paper we report: (1) the application of gene-based markers in faba bean; (2) the development of the first exclusively gene-based genetic and comparative map for the species; (3) analysis of syntenic relationship between faba bean and *M. truncatula*; and (4) the levels of homology existing between faba bean, *M. truncatula *and lentil, a closely related species to faba bean.

## Methods

### Genetic mapping population

A gene-based genetic map of faba bean genome was constructed using a population of 94 F_6 _RILs generated from a cross between faba bean line Vf6 (*equina *type) as the pollen recipient and line Vf27 as pollen donor (*paucijuga *type). These accessions have been widely used in previous genetic studies [7,9,21] and the population was developed at IFAPA, Centro Alameda del Obispo, in Córdoba, Spain, using diploid parental individuals. Total genomic DNA was isolated from each parent and F_6 _individual as previously described [22].

### Primer design

A total of 796 intron-targeted amplified polymorphic markers (ITAPs) were used for this study. These were composed of four sets of ITAPs; 340 ML and Lup primers developed from alignment of *M. truncatula *and *Lupinus *spp. database EST sequences; 160 MLG primers based on alignments between *M. truncatula*, *Lupinus albus*, and *Glycine max *([15], with a subset of these denoted AtMtL-); 143 cross-species makers (MP) developed by the Department of Plant Pathology, University of California, Davis, USA [12]; and 140 GLIP markers created by the European Grain Legumes Integrated Project (GLIP) based on primarily on *M. truncatula *and pea (Andrea Seres, pers. comm.). The majority of the ITAPs markers could be positioned to a physical location in the *M. truncatula *psuedogenome since most primers were designed from genes in characterized chromosomal regions.

### Polymorphism detection

Each primer pair was screened on *V. faba *parental DNA. PCR conditions were optimised to produce clear single amplicons, and single PCR products of the same size were purified and directly sequenced. Different detection methods were used to genotype the F_6 _population dependent on the type of polymorphism [15]. Details of each marker are given in Additional file 1.

### Map construction

Chi-squared analysis (P < 0.05) was applied to test the segregation of the mapped markers against the expected Mendelian segregation ratio for co-dominant inheritance in the faba bean F_6 _RIL population. Genetic linkage mapping was conducted with MultiPoint v 1.2 software [23], with a recombination fraction (rf) of 0.29 (LOD = 9.0) using 5000 bootstraps. Map distances were calculated in cM by applying the "Kosambi" function. Groups of linked markers that were similarly distorted were accepted for linkage mapping. Independent markers showing significant segregation distortion and markers with missing data (> 10%) were rejected for linkage to avoid bias and false linkages. Genetic maps were drawn with the software program MapChart v 2.1 [24].

### Establishment of macrosyntenic relationships between faba bean, lentil and *M. truncatula*

Markers mapped in faba bean were located on the *M. truncatula *map by aligning the ESTs originally used to design the ITAPs primers with *M. truncatula *BACs in the Medicago pseudogenome Mt2.0 build 8/10/2007. Alignments with a BLAST E values < 1e^-20^, hsp identity ≥ 60%, and hsp length > 50 nt were retained. Precise positions of markers were obtained by aligning the ITAP primer sequences with *M. truncatula *BACs using BLASTN with an expected value < 1e^-4 ^(primer length varied from 18bp to 28bp). Approximate positions of markers that had been genetically mapped in *M. truncatula *but not yet positioned on the physical map were obtained from the *M. truncatula *genetic map [25].

### Loci dot plot created via Grid Map

Grid Map [26] was used to compare the genetic maps of faba bean and *M. truncatula*. Ordered loci from faba bean and *M. truncatula *linkage groups were listed vertically and horizontally, respectively, and dots were positioned on the diagram at the intersection of the locations of the corresponding markers in the two genetic maps.

## Results

### Gene-based marker development

Of the 796 markers screened for amplification in genomic faba bean DNA, 19% produced two or more amplicons of different sizes. Five hundred and seven were selected and optimised for single-locus amplification. Fifty percent (254) of these produced clear single band amplicons (Table 1). Seven markers that produced two amplicons (AIGP, GLIP172, GLIP429, GLIP621, GLIP651, Lup226 and MMK1) and one that produced three amplicons (LG34) were also mapped as they exhibited convenient length polymorphisms. One hundred and sixty-five polymorphic markers were identified (Table 1), of which 151 markers were used to genotype the 94 individuals of the F_6 _RIL population (supplementary Table 1). Apart from the GLIP markers which were selected for their ability to amplify faba bean genomic DNA before this study, 'MLG' and 'MP' markers worked equally well in faba bean (83% and 84%, respectively) but 'MP' markers produced fewer polymorphic amplicons. Amplification rate of the 'ML' markers was the poorest (34%), less than half the rate of the 'MLG' and 'MP' groups (Table 1). Likewise, the polymorphism level of the 'ML' markers was the lowest. Both 'MLG' and 'GLIP' markers produced very high levels of polymorphism in the mapping parents (70%; Table 1). DNA sequences of markers have been submitted to Genbank under accession codes FH893713 – FH937528.

**Table 1:** Efficiency of gene-based markers used to construct the comparative genetic linkage map of faba bean

Marker type	Screened	Amplification^a^	Length polymorphism	Sequenced^b^	CAPS/SNP^c^	Mapped
MP	143	120 (84%)	6	75 (63%)	45 (60%)	36
ML	340	115 (34%)	9	30 (26%)	16 (53%)	25
MLG	160	132 (83%)	5	73 (55%)	50 (70%)	42
GLIP	140	140	6	76 (54%)	54 (71%)	48

Total	796	507	26	254	165	151

^a^Figures in parentheses are percentages of amplified markers of the total markers screened.

^b^Figures in parentheses are percentages of sequenced markers of the total amplified markers.

^c^Figures in parentheses are percentages of polymorphic markers of the total sequenced markers.

**Figure 1:**
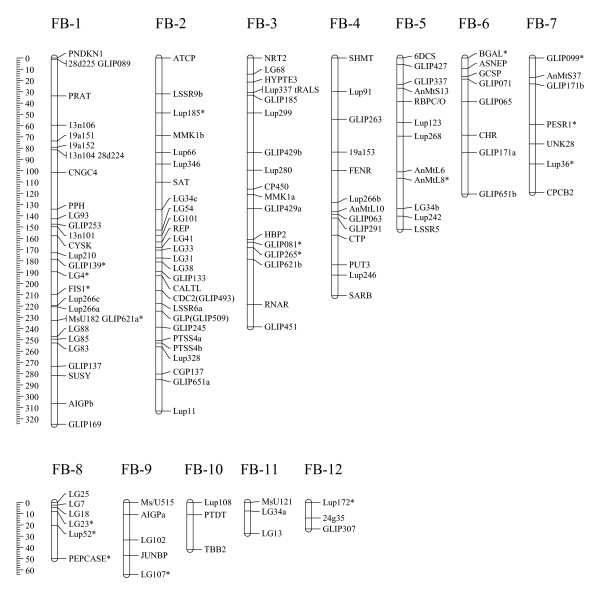
**A gene-based genetic linkage map of faba bean (*Vicia faba *L)**. Marker distance is given in cM. *indicates markers with distorted segregation.

Nineteen mapped markers (> 12%) deviated significantly (P < 0.05) from the expected Mendelian inheritance ratio of 1:1. About half of these markers (9) were highly distorted (P < 0.01). Fifty percent of the distorted markers segregated in favour of the Vf6 parent and fifty percent in favour of Vf27. Nine of the 12 faba bean LGs contained one to four of these distorted markers, which tended to be scattered throughout the faba bean genome. However, it is noteworthy that distorted markers grouped together in chromosomes FB-1, 7 and 8 (Figure 1).

### The first gene-based and comparative map of Faba bean

A total of 151 genic markers were used to generate the first gene-based and comparative map of faba bean. This map was constructed with a recombination fraction of 0.29 [LOD = 9.0, 27]. The map consists of seven main linkage groups (FB-1 to FB-7) and 5 fragments (FB-8 to FB-12), which varied in length from 23.6 to 324.8 cM, and spans a total of nearly 1686 cM (Figure 1 and Table 2). The number of markers per LG ranged from three to 30 markers. In addition, there was one pair and thirteen unlinked markers. Eight markers co-segregated at four loci, (three loci in FB-1, one in FB-3; Figure 1 and Table 2). The maximum distances between markers ranged from 13.8 cM in FB-12 to 40.2 cM in FB-3 with an overall mean gap distance of 14.6 cM (Table 2).

**Table 2:** Properties of the faba bean comparative map

Linkage group	Length of LGs (cM)	No of markers	No of loci	Average marker spacing^a ^(cM)	Largest distance between markers (cM)
LG-1	324.8	30	27	12.5	32.5
LG-2	313.1	27	27	12.0	31.8
LG-3	238.5	18	17	14.9	40.2
LG-4	210.7	13	13	17.6	29.8
LG-5	151.9	12	12	13.8	26.8
LG-6	120.7	8	8	17.2	36.9
LG-7	119.2	7	7	19.9	35.9
LG-8	49.9	6	6	10.0	29.5
LG-9	64.1	5	5	16.0	22.6
LG-10	41.8	3	3	15.4	30.7
LG-11	27.5	3	3	13.8	19.9
LG12	23.6	3	3	11.8	13.8

Total	1685.8	135	131		

^a^Calculated by dividing the length of the chromosome (cM) by the number of space/distance between markers/loci.

**Figure 2:**
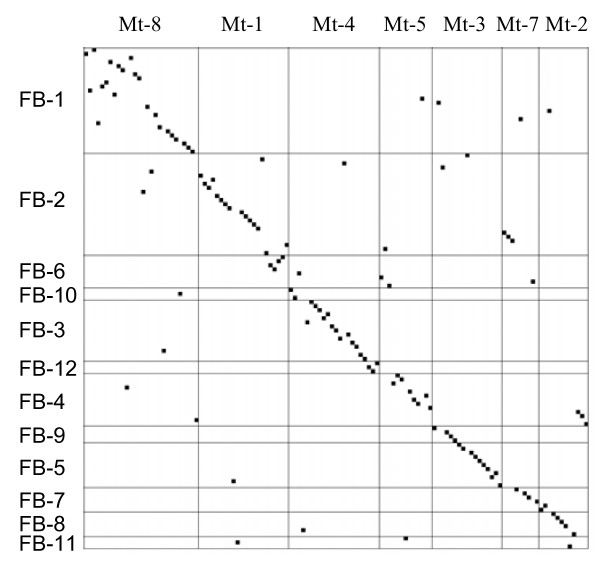
**Matrix plot of common gene-based markers mapped in faba bean and *M. truncatula***. The faba bean and *M. truncatula *loci are listed vertically and horizontally, respectively, according to their linkage group order.

Of the 135 genic markers that mapped to the 12 faba bean LGs in Figure 1, 127 were assigned to the *M. truncatula *genetic or physical maps [25]. One hundred and four of these (82%) were in syntenic regions. Clear evidence of a simple and direct macrosyntenic relationship between the *V. faba *and *M. truncatula *is presented in the dot matrix in Figure 2. The formation of clear isoclinic diagonal lines along the linkage groups provides a strong indication of the conservation of gene order in the two legume genomes. The extensive colinearity was particularly prevailing between FB LG 1, 2, 3, 4 and Mt LG 8, 1, 4, 5 where syntenic regions accounted for 90%, 86%, 50% and 47% of the *M. truncatula *pseudogenome, respectively (Table 3).

**Table 3:** Colinearity between the faba bean and *M. truncatula *genomes

FB LG	No. of markers	No. of non-orthologous markers	No. of unassigned markers	Current Mt genetic coverage (LG: cM, No. colinear markers)^1,2^	Current Mt pseudogenome coverage (bp)^1^	Current faba bean genetic coverage (cM)^2^
FB-1	30	6	2	8: 0 – 68.2, 11	8: 736241 – 32433863 (90%)	0 – 324.8 (100%)
FB-2	27	4	2	1: 0 – 58.5, 9 7: 58.4 – 60, 3	1: 59849 – 26963044 (86%) 7: 25725918 – 26383168 (2%)	152.6 – 313.1 (51.3%) 157.1 – 210.7 (25.4%)
FB-3	18	1	3	4: 48.1 – 59, 11	4: 14544831 – 34128326 (50%)	0 – 238.5 (100%)
FB-4	13	2	0	5: 0 – 32, 6 2: 12.7 – 37.9, 3	5: 594897 – 17960204 (47%) 2: 8491236 – 15201671 (24%)	0 – 142 (67%) 250.5 – 256.3 (1.9%)
FB-5	12	1	1	3: 28 – 70.3, 9	3: 10888141 – 28213807 (46%)	0 – 140.3 (92%)
FB6	8	4	0	1: 2 – 2.2, NA	1: 4942324 – 5151182 (0.7%)	-
FB-7	7	0	0	7: 22.6 – 52.8, 4	7: 9999559 – 22150020 (38%)	0 – 58.9 (49%)
FB-8	6	1	0	2: 48 – 57.8, 5	2: 17523205 – 20646361 (11%)	0 – 49.9 (100%)
FB-9	5	0	0	3: 62 – 72.5, 5	3: 19000000* – 30712150 (31%)	0 – 64.1 (100%)
FB-10	3	1	0	4: 0 – 7.4, NA	4: 537530 – 3489843 (8%)	-
FB-11	3	3	0	NA	NA	-
FB-12	3	0	0	4: 60.4 – 61.1, NA	4: 36664007 – 38186081 (4%)	-

NA: not applicable

^1^Data was either based on information from *M. truncatula *genome sequencing website [25] or from Choi *et al*. [12].

^2^Data is provided for three or more colinear markers and excludes markers on the same chromosome rearranged relative to the order in *M. truncatula*.

However, chromosomal rearrangements were also evident at a moderate level. For example, *M. truncatula *chromosomes 1 and 7 together with 5 and 2 merged to form the faba bean LGs 2 and 4, respectively. Similarly, *M. truncatula *chromosome 2 splits into FB- 4 and 8 and *M. truncatula *chromosome 3 into FB-5 and 9 (Table 3 and Figure 2). Inversions and translocations were also notable among the orthologous markers within each syntenic pair of faba bean and *M. truncatula *LGs (Figure 2).

### Evidence of macrosynteny among faba bean, lentil and Medicago truncatula

**Figure 3:**
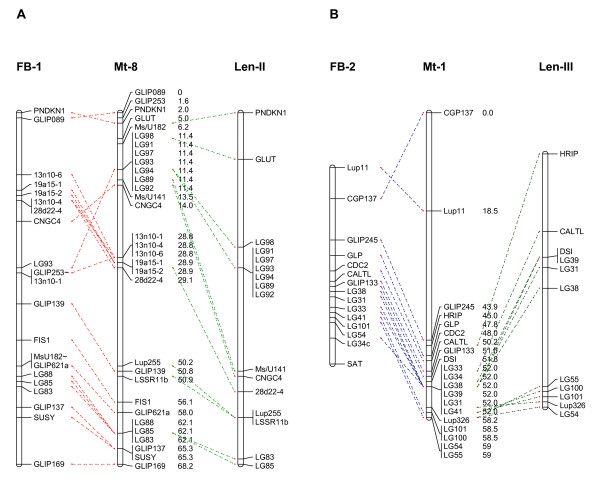
**Evidence of shared macrosynteny between *V. faba *chromosomes FB-1 and FB-2, *L. culinaris *and *M. truncatula***. Common orthologous markers are depicted by dashed lines and marker distances are provided in centi-Morgans for *M. truncatula *only. The figures exclude markers that could not be positioned in the *M. truncatula *psuedogenome. A ~ indicates markers that map distally in the corresponding *M. truncatula *chromosome relative to faba bean, and markers suffixed a, b or c denotes derivation from primer pairs that produced multiple PCR products.

**Figure 4:**
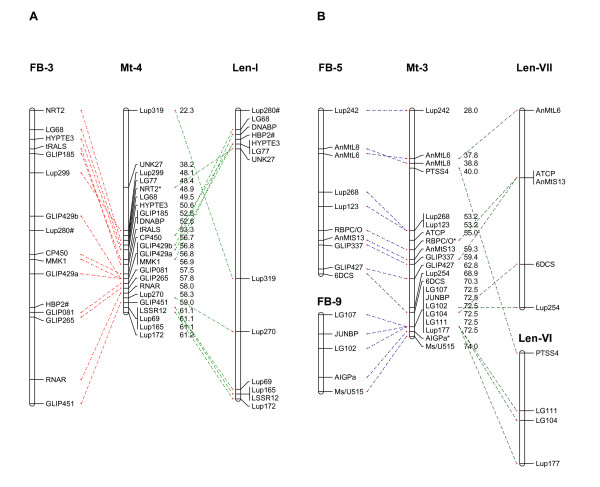
**Evidence of shared macrosynteny between *V. faba *chromosomes FB-3 and FB-5, *L. culinaris *and *M. truncatula***. Common orthologous markers are depicted by dashed lines and marker distances are provided in centi-Morgans for *M. truncatula *only. The figures exclude markers that could not be positioned in the *M. truncatula *psuedogenome. Markers highlighted * were previously mapped [18] but not positioned in the *M. truncatula *psuedogenome. # indicates markers orthologous between lentil and faba bean but unmapped in *M. truncatula *and markers suffixed a, b or c denotes derivation from primer pairs that produced multiple PCR products.

A high level of co-linearity was found between the faba bean, lentil and *M. truncatula *genomes based on the macro-synteny established between faba bean and *M. truncatula *(this study) or lentil and *M. truncatula *[15] using only common orthologous markers which mapped in all three species (Figures 3 and 4). The pattern of homology between faba bean and *M. truncatula *was similar to that between lentil and *M. truncatula*: for example, two linkage groups FB-1 and Len-II were exclusively syntenic to *M. truncatula *LG_8 and shared seven markers in common. Common markers were evenly distributed in all the three corresponding LGs suggesting that FB-1 and Len-II are essentially co-linear (Figure 3A). Likewise, FB-2 and Len-III were both syntenic to Mt-1 and orthologous to each other with nine markers in common (Figure 3B). Other examples are FB-3, Len-I and Mt-4; and FB-4, Len-V and Mt-5 (Figure 2, 4A and Phan et al., 2007 [15]). FB-5 and FB-9 were co-linear with lentil Len-VII and LenVI, respectively, and both pairs of these linkage groups were colinear with Mt-3 (Figure 4B). This suggests shared ancestral chromosomal changes in faba bean and lentil compared to *M. truncatula *and confirms their phylogenetically closer relationship.

### Evidence of fine scale macrosynteny on individual BACs between faba bean, lentil and Medicago truncatula

Several ITAP markers used in this study were designed from different genes on the same *M. truncatula *BACs. These markers often mapped in clusters in two or more species and in common syntenic regions of corresponding linkage groups. An example is shown in Figure 3A with markers LG89-LG98 designed from *M. truncatula *BAC AC140032 and LG83-LG88 from *M. truncatula *BAC AC138131 in *M. truncatula *chromosome 8. Similarly, with the exception of LG34c, markers LG31-LG43 designed from *M. truncatula *BAC AC152751 in Mt chromosome 1 were located in one area of FB-2 and Len-III as shown in Figure 3B; markers AnMtL6-AnMtL8 and LG102-LG112 from *M. truncatula *BAC AC147712 and AC135800 in Mt chromosome 3 were grouped together on corresponding pairs of LGs i.e. FB-5 and Len-VII and FB-9 and Len-VI; respectively (Figure 4B).

## Discussion and conclusion

The first genetic map of faba bean composed exclusively with gene-based co-dominant molecular markers was constructed using a F_6 _RIL population between lines Vf6 and Vf27. The map is also the first to enable the establishment of syntenic relationships between faba bean and the model legume *M. truncatula*, comparison with other legume species, and integration with genetic maps available in faba bean.

The map is composed of 12 linkage groups and 151 genetic markers. Although the number of chromosomes in faba bean has been reported as 2n = 12 [2], the number of linkage groups in recent genetic maps in the species range from 13 to 18 [7-10] and previously as many as 48 have been reported [6]. The high number of linkage groups compared to the number of chromosomes may be due to the fact that faba bean possesses one of the largest genomes among cultivated legumes (~13000 Mb). This compares with other well-characterised species such as *M. truncatula*, chickpea, soybean, lentil and pea which have genomes of ~450 Mb, ~740 Mb, ~1200 Mb, ~4000 Mb and ~4000 Mb respectively [28].

Of the 24 non-orthologous markers found in this study, eight were from primer pairs where more than one PCR gel band was present and where two or more such amplicons were mapped. In each case, at least one amplicon mapped syntenically. The percentages of markers sequenced in faba bean were lower compared to lentil (63%, 26% and 55% compared to 93%, 69% and 65% for MP, ML and MLG markers respectively, Table 1). This was due to higher proportion of markers amplifying multiple bands in faba bean compared to lentil (data not shown), which may imply duplication. Differences in amplification, sequencing and polymorphism rates among different types of markers used for this study reflect the mode of design of the markers. Since 'MP' and 'MLG' markers were often based on the homology of more than two phylogenetically distant species, they are more likely to work in different legume lineages. The same observation was reported for these primer sets in lentil [15].

Despite the large differences in genome sizes between *M. truncatula *and *V. faba*, a simple and direct relationship between the two genomes was identified in this study. Given the number of markers used (151), the syntenic regions cover a large proportion of *M. truncatula *pseudogenome with 90%, 87%, 66%, 62% and 47% for *M. truncatula *chromosomes 8, 1, 3, 4 and 5, respectively (Table 3). The appearance of clear isoclinic diagonal lines along the linkage groups in Figure 2 also demonstrates strong evidence for the extensive co-linearity between linkage group pairs of the two species. Similar high levels of conservation have also been reported between *L. culinaris *ssp. *culinaris *and *M. truncatula *[15] and other closely related legumes such as *L. culinaris *ssp. *culinaris *and *P. sativum *[29], *M. sativa *and *P. sativum *[30], *M. truncatula *and *P. sativum *[17], *M. truncatula *and *M. sativa *[11]. This study also shows markers originally designed from genes on the same BAC clustered in corresponding syntenic areas in lentil and faba bean. The mapping populations were too small to resolve marker order in lentil and faba bean but extensive conservation of gene order,(and microsynteny) has been shown in previous studies between other legume species at similar or greater phylogenetic distances [12,31-34], and to some extent between *M. truncatula *and *Arabidopsis *[33-35].

A higher level of homology between *V. faba *and *L. culinaris *ssp. *culinaris *compared to that between *V. faba *and *M. truncatula *could be inferred from this study based on the common markers mapped in the two genomes, common homology with *M. truncatula *and similar pattern of rearrangements (Figures 3, 4 and Phan *et al*., 2007 [15]). This finding agrees with phylogenetic studies that place the genera *Vicia*, *Lens *and *Pisum *within the tribe Viceae while *Medicago *and *Melilotus *form a parallel tribe Trifolieae within the Galegoid or cool season legumes [36], and is consistent with different levels of macrosynteny observed between *M. truncatula*, *P. sativum*, *V. radiata*, *G. max*, and *Phaceolus vulgaris *dependent on phylogenetic distance [12]. However, chromosomal rearrangements were evident (Figures 2 and 3).

Rearrangements involving Mt6 and Mt3 in particular may explain the differences in chromosome number between the two species (*M. truncatula*: n = 8; *V. faba*: n = 6). Mt6 might be considered unusual and is largely composed of heterochromatic DNA [37], contains few transcribed genes [11] and a large proportion of resistance gene analogues [38]. In this study no corresponding linkage group was detected in faba bean, as found previously in pea [12] and lupin [13,14], together with less than five percent estimated coverage by the *L. japonicus *genome [34]. In faba bean FB5 and FB9 appear to correspond to Mt3. This configuration is supported by a similar pattern in lentil (Figure 4B and Phan *et al*., 2007 [15]), although a larger number of markers are needed to confirm this.

The faba bean comparative map constructed here is consistent with the pattern of chromosome conservation previously observed, where different levels of conservation were found to be relatively consistent between *M. truncatula *and other legume species i.e. high conservation of *M. truncatula *chromosomes 1, 5 and 8; moderate conservation in the *M. truncatula *chromosomes 2, 3, 4 and 7 and lowest conservation in the *M. truncatula *chromosome 6 (Figures 3, 4 and [39]). As described above, no homology was identified with *M. truncatula *chromosome 6 in this study. The alignment of this faba bean map with lentil and the current *M. truncatula *genome based on *M. truncatula *genome assembly Mt2.0 is slightly different to that based on an earlier assembly [15]. The changes can be observed in Figure 3B where orthologous markers which were syntenic to *M. truncatula *chromosome 6 in lentil are now co-linear with *M. truncatula *chromosome 1 in common with faba bean.

Genome studies have demonstrated different factors are responsible for genome size variation and speciation. These include ancient polyploidisation events in the case of the Brassicas [40]; segmental or region-specific duplication [41]; and genetic rearrangements, transposable element amplification, or combination of different genome modifications [42]. Large scale rearrangements, duplications, or polyploidisation were not apparent in this study, possibly as a result of the focus on single locus markers, however differences in non-coding repetitive DNA or transposable elements provide a possible explanation for the large differences in genome size. Retroelements are known to account for substantial proportions of these Viceae genomes as shown by extensive studies in pea, for example [43,44], and more recently *Vicia *[45,46]. Local genic rearrangements similar to that in found in the grasses (duplications, translocations, and insertions or deletions) may explain multiple PCR amplicons [19,20].

The shared macrosynteny among the three species demonstrated here and even higher level of homology between *L. culinaris *and *V. faba *will undoubtedly facilitate the identification of markers closely linked to traits of interest in *V. faba*. Alignment of this map with existing faba bean maps containing important traits with polymorphic SSR markers and/or markers developed in this study, coupled with cross-reference to the abundant genetic information from the Medicago genome sequencing and extensive EST libraries available for the model legume species, will undoubtedly assist this process. As the parental line Vf6 has been used in a number of genetic and QTL mapping projects [6,7,9], this map can serve as a central reference map. This study has provided a number of significant outcomes for faba bean genomics and legume genomics in general.

## Authors' contributions

SE and RPO designed the research. HTTP and SE wrote the manuscript. HTTP, SE and MG performed marker polymorphism discovery and population genotyping in Perth. JH assisted with informatics procedures. AMT, CMA and SC-I were involved in developing recombinant inbred lines and marker polymorphism discovery of GLIP markers in Córdoba. All authors read and approved the final manuscript.

## Supplementary Material

Additional file 1 Supplementary table 1. Orthologous PCR markers developed in this study and genetically mapped in a F_6 _faba bean population between lines Vf6 and Vf27. Click here for file
