# Supplementary material for: Construction of a comparative genetic map in faba bean (Vicia faba L.); conservation of genome structure with Lens culinaris
Source: BMC Genomics. 2008 Aug 9;9:380. doi: 10.1186/1471-2164-9-380 (PMC2533332; doi:10.1186/1471-2164-9-380)
Supplement: Additional file 1 — Supplementary table 1. Orthologous PCR markers developed in this study and genetically mapped in a F6 faba bean population between lines Vf6 and Vf27. [file 1471-2164-9-380-S1.doc]

**Supplementary table 1**: Gene-based, PCR markers used in this study and genetically mapped in faba bean using an F6 RIL population developed from lines Vf6 and Vf27

| Marker name | Type | Template GenBank accession number | Sequenced amplicon accession number (Vf6) | Putative function | Assay method | Restriction enzyme, SNaPshot primer, or internal SNP primers | Vf 6 band sizes or SNaPshot SNP | Vf 27 band sizes or SNaPshot SNP | Forward primer sequence | Reverse primer sequnce |
| --- | --- | --- | --- | --- | --- | --- | --- | --- | --- | --- |
| 13n10-1 | MLG ITAP | bi968243 | FH893857 | Multidrug resistance-associated protein-like protein [Vitis vinifera]. | SNaPshot | TCCACCACTTTCCCTTTAATAA | T | C/64/F | GCAGCTCGACCAATATTCAGC | GATAGATAGCGACATGGTCATGG |
| 13n10-4 | MLG ITAP | bi785323 | FH893856 | Retinoblastoma-related protein [Medicago sativa]. | CAPS | BstBI | 110+230 | 340 | ATGAGTTGAGGAAAACCATGGAG | CTGTCATCCTCATTAATGAATAACC |
| 13n10-6 | MLG ITAP | tc208498 | FH893854 | similar to [*UP|Q9M5G5*](http://www.pir.uniprot.org/cgi-bin/upEntry?id=Q9M5G5) (Q9M5G5) Retinoblastoma-like protein (Fragment) | CAPS | TaqI | 170+30 | 200 | GTTGGGGAAAGCACTCATGC | CTGCAGATAGAGGCTGTTTGC |
| 1433P | Mt cross-species | AI974411 | FH893853 | Homologue to UP|Q9T0N0 (Q9T0N0) 14-3-3-like protein, complete | CAPS | FokI | 530 | 510+20 | AAGGTTTTCTACCTTAAGATGAAGGGAG | GTTTAGCAAGATTGCAGGCACGA |
| 19a15-1 | MLG ITAP | tc206234 | FH893843 | Homologue to UP|Q93XA1 (Q93XA1) TGA-type basic leucine zipper protein TGA2.1, partial (76%) | CAPS | MboII | 210+130 | 340 | ACTTCGCATGATTGTTGATGG | ATACCCATCAGCTGCTGCTC |
| 19a15-2 | MLG ITAP | tc206234 | FH893850 | Homologue to UP|Q93XA1 (Q93XA1) TGA-type basic leucine zipper protein TGA2.1, partial (76%) | CAPS | NlaIII | 220 | 180+40 | CAAATTACATGGGTCAAATGGC | AGGAGTGCACGAGCTGATTG |
| 19a15-3 | MLG ITAP | tc230915 | FH893848 | Similar to [*PIR|T00862*](http://pir.georgetown.edu/cgi-bin/nbrfget?xref=1&id=T00862) probable serine/threonine-specific protein kinase F17K2.2 - Arabidopsis thaliana | SNaPshot | ATCCGAAGAAACAAGAGGG | A/127/R | C | GGTGCTCCTCCATTTGAGGC | CAAGGATGCTCCATTATCTTCTG |
| 24g3-5 | MLG ITAP | tc226851 | FH893847 | Similar to UP|Q84WJ9 (Q84WJ9) At5g19680, partial (47%) | SNaPshot | AGCCGGAGATTGACTAAAGA | T/26/R | C | GCTCTAGAAGAACTGTACTTGAGCC | AGACTGCCTCAGCAATTCCTTC |
| 28d22-4 | MLG ITAP | tc207024 | FH893871 | Similar to UP|Q93ZI1 (Q93ZI1) AT3g12300/F28J15_117, complete | CAPS | XhoI | 300 | 190+110 | CCAACTCTTTTCAAGGGCAGC | TGTCCCAAGCTCCAATTTCC |
| 28d22-5 | MLG ITAP | bg156340 | FH893869 | Similar to TR:Q9SGV5 Q9SGV5 F1N19.17. | CAPS | XhoI | 300 | 190+110 | CCAACTCTTTTCAAGGGCAGC | TGTCCCAAGCTCCAATTTCC |
| 6DCS | Mt cross-species | AW126337 | FH893864 | NAD(P)H-dependent 6-deoxychalcone synthase | CAPS | HpyCH4IV | 380 | 200+180 | CTCACGAAAGCCATTGGAGCCAGCAACT | TGCCATGAGCATCAGCCAATTCTTTGA |
| AIGPa | Mt cross-species | AW125928 | FH893940 | Auxin-independent growth promoter | SNaPshot | CAGCCATGCGAGAAGAAAA | C/195/F | G | CTGATAGGGCCAGGAGGCAGGGAAGA | GTTTTTTAGCATTTGGACGAATGGTTGGT |
| AIGPb | Mt cross-species | AW125928 | NA | Auxin-independent growth promoter | Length | NA | 300 | - | CTGATAGGGCCAGGAGGCAGGGAAGA | GTTTTTTAGCATTTGGACGAATGGTTGGT |
| AnMtL10 | MLG ITAP | CA410800 | NA | nucleic acid binding [Arabidopsis thaliana]. | Length | NA | 540 | 580 | CCCGGTTACGAATCTTCGCATC | GATCTAGAGTTGGATTTTGCAAGATC |
| AnMtL6 | MLG ITAP | CA410275 | FH893826 | ATP binding / kinase/ uracil phosphoribosyltransferase/ uridine kinase | CAPS | DdeI | 300 | 210+90 | CAAGAGTACAAGATTACAACTTTGACC | CTTTGGAATATCTACTGCTTCGCC |
| AnMtL8 | MLG ITAP | CA410275 | FH893827 | Uracil phosphoribosyltransferase, putative / UMP pyrophosphorylase | SNaPshot | TCTAGTTATTTTCCAATCCGGTAG | A/245/F | G | CGTGTTCGGGAGTTGATGAATATG | CCTAATCCTTCTAGCTAAACGAACATC |
| AnMtS13 | MLG ITAP | AC145219 | NA | Carboxylic ester hydrolase activity | Length | NA | 510 | 430 | GCGACGTGCCCTCCAAGTCC | AGGACCCATGGAATCATTACCTCC |
| AnMtS37 | MLG ITAP | TC207479 | FH893829 | Homologue to UP|KDSA_PEA (O50044) 2-dehydro-3-deoxyphosphooctonate aldolase (Phospho-2-dehydro-3-deoxyoctonate aldolase) | CAPS | AvaII | 370+380 | 750 | GATTCCAGCATTCTTGTGTCGCC | CATCCACTCCAAGTTACGTGGATC |
| ASNEP | Mt cross-species | AW208187 | FH893831 | Similar to UP|VPE_VICSA (P49044) Vacuolar processing enzyme precursor (VPE) Proteinase B, partial (95%) | CAPS | HindIII | 640 | 120+520 | GAAGAAAAAACATGCTTCTGGATCATATA | ATCTTTAACCAATTTATATTGCTGGTGCA |
| ATCP | Mt cross-species | AW207998 | NA | Aquaporin-like transmembrane channel protein | Length | NA | 130 | 220 | AACCAATTGGTATTGCAGCTCAGAGCCA | TTCCTTGCCAAGAACAAACCGAATGTCA |
| BGAL | Mt cross-species | AW126229 | FH893832 | Beta-galactosidase | CAPS | HpaII | 510+50 | 560 | AGACTTGTCTTGGCAGAAATGGTCTTACA | AATATGATACCATTTCTGTGTTGGTTCTCCA |
| BGER | Mt cross-species | AW191286 | FH893834 | Beta-glucan-elicitor receptor | CAPS | DdeI | 520 | 280+240 | TGGTGATTTCGGTTTCGGAATTTACAATGA | CACTTTCATTATCATAAATTCCTTCCAATGA |
| CALTL | Mt cross-species | AW126242 | FH893835 | Calreticulin | SNaPshot | GGTATTGAATTGTGGCAGGTAA | G/140/F | A | GTGGAAGGCACCATTGATTGACAAC | TCTTCTTCTCAGCCTCTTCAAATGC |
| CDC2 | Mt cross-species | AW171750 | FH893836 | Putative cdc2 kinase | CAPS | DdeI | 700 | 430+270 | CAACTTTGCAAGGGTGTTGCTTTCT | ACTAACACCTGGCCACACATCTTCA |
| cgP137F | Mt cross-species | AW257467 | FH893838 | Unknown protein | CAPS | HpaII | 260+170 | 430 | TGTTAACCTCAGTTACTGCAACAGA | AGGATGCAATCAAGCATATATCTTGA |
| CHR | Mt cross-species | AI974694 | FH893868 | Chalcone reductase | CAPS | AvaI | 270 | 210+60 | TTTGGGTTACTGAAAATCATCCTCATCTTG | GAGAGAAGATTTTCAAGTTTCTTGACAGA |
| CNGC4 | Mt cross-species | AW126067 | FH893839 | Cyclic nucleotide gated channel -like protein | SNaPshot | TCACATGTAAACAAGTATAGTTAAGTGAA | T/217/R | G | AGAGATGAGAATCAAGAGGAGGGATGCA | CATGATGAAGAGCATTTCGTCCACTGGA |
| CP450 | Mt cross-species | AW171693 | FH893844 | Cytochrome P450 | CAPS | AvaII | 330 | 310+20 | AGTGTGAGATCAATGGTTATGTGATC | CATCATCACCTTTCAATATTTGTCC |
| CPCB2 | Mt cross-species | AW191283 | NA | Putative coatomer protein complex, subunit beta2 | Length | NA | 620 | 640 | AGAAAGAGTGAAGTCTGTGGATCTACATC | GGATGAACAGCCACACACCTAATGTAATC |
| CTP | Mt cross-species | AW126130 | FH893866 | Putative carboxyl-terminal peptidase | CAPS | AciI | 200+140 | 340 | GTTTCGACCCGGACCACCATAATAGAAGTA | TGGAAATGACCATGTTCTAGGATACTGGC |
| CULL | Mt cross-species | AW127073 | FH893841 | Putative cullin protein | CAPS | MseI | 390 | 280+110 | GCAGAGTTGATTGCCAAGTTTTTGGATGA | CCTTAAACATTCCTTCCAGCTTGTTTGTA |
| CYSK | Mt cross-species | AW207985 | FH893842 | Cystein synthase (O-acetylserinesulfhydrylase) | SNaPshot | GGCCATGTTCCAGTGTTAAA | A/100/F | G | GGAATTGCTAAAGATGTTACAGAATTGA | AATGAGGACACTCTGTCCAGGTGTGA |
| EXRN | Mt cross-species | AI974855 | FH893924 | Putative exoribonuclease | Length | NA | 750 | 770 | GTTGGTGGTGGAAGTGATGGATCTCTGGA | AACCTAGATATGTTCGGGTAAGATACTTGA |
| FENR | Mt cross-species | AW127593 | FH893921 | Ferredoxin-NADP reductase precursor | CAPS | SacI | 700 | 590+110 | ATGCTTATGCCAAAAGATCCAAATGC | ATCTTGTCACCGGGATCAAGCACACA |
| FIS1 | Mt cross-species | AI974522 | FH893919 | fis1 protein | CAPS | SspI | 580 | 440+140 | TTGCCACAGTAGAAGCAGAAGAGATA | CTGTTTCATCAACTTCAGCAACTTT |
| GCSP | Mt cross-species | AW127448 | FH893863 | Glycine dehydrogenase (decarboxylating), mitochondrial precursor | CAPS | HphI | 600 | 500+100 | GTAAAGAAACACTTGGCACCATTTTTAC | CCTCTTAAGTCAATAATGAACTCATGAGC |
| GLIP063 | GLIP ITAP | TC96372 | FH893904 | Similar to UP|Q75KB9 (Q75KB9) Expressed protein, partial (83%) | CAPS | SfcI | 230+50 | 280 | TCTTCAGGAGGTAGCTGGGA | CAAGGCGAAGAAAGAACGAC |
| GLIP065 | GLIP ITAP | TC94522 | FH893907 | Similar to UP|Q84TF0 (Q84TF0) At2g37790, partial (96%) | CAPS | AciI | 570 | 280+290 | CAGAAGATGTGCCAAAAGCA | GCTTCCAATGCTTTCCATGT |
| GLIP071 | GLIP ITAP | TC106695 | FH893906 | Homologue to UP|GCSP_PEA (P26969) Glycine dehydrogenase [decarboxylating], mitochondrial precursor (Glycine decarboxylase) (Glycine cleavage system P-protein) , complete | SNaPshot | ACTCACTCCGTCTCATAGTAACTGT | T/123/F | C | TTTGCCACAGTATGATCCCA | CAGCATATTCTCCAGCAGCA |
| GLIP081 | GLIP ITAP | TC111019 | FH893884 | Similar to [UP|Q6X0N7](http://www.pir.uniprot.org/cgi-bin/upEntry?id=Q6X0N7). Putative beta-galactosidase | CAPS | HindIII | 440 | 200+240 | GTCAAGCTTGGGTGAATGGT | TTGGATGCTTTCAACCATGA |
| GLIP089 | GLIP ITAP | CX535064 | FH893882 | Similar to [UP|Q94LQ9](http://www.pir.uniprot.org/cgi-bin/upEntry?id=Q94LQ9) (Q94LQ9) Putative transcription regulatory protein | SNaPshot | TGGTCTCATGCCCAATAGTTCT | C/140/F | T | CTTCATGCCAAAAATGCTGA | GAAGAAGCACCAAGAGCACC |
| GLIP099 | GLIP ITAP | TC95536 | FH893873 | Similar to UP|Q9FYW9 (Q9FYW9) BAC19.7, partial (54%) | CAPS | MaeIII | 20+63+317 | 380+20 | ACAAGCAACCATGTTGGACA | GAGCAGATCACCTCCTGGAC |
| GLIP107 | GLIP ITAP | TC101640 | FH893918 | Similar to UP|Q9FR37 (Q9FR37) Amidase (At1g08980/F7G19_15), partial (80%) | CAPS | AciI | 540+140 | 680 | CTGATTGGGCAAGGACTCAT | AAATCCACAAGCTTTGCACC |
| GLIP133 | GLIP ITAP | TC108333 | FH893916 | Similar to unknown protein {Arabidopsis thaliana;, partial (68%) | CAPS | TaqI | 610+190 | 800 | AAGATGGAGGTTGATGTGCC | ACTTCAAGGACCATTGCGAC |
| GLIP135 | GLIP ITAP | BI272042 | FH893879 | Homologue to UP|Q84XA4 Aminoimidazolecarboximide ribonucleotide transformylase/inosine monophosphate cyclohydrolase, partial (21%) | SNaPshot | TTTTTTGCGGGCGCA | A/165/R | G | CCGAATCGTGTGGAGAGTTT | TCACATGCTTCTTCCACAGC |
| GLIP137 | GLIP ITAP | TC101045 | FH893912 | Homologue to UP|Q5ZP80 (Q5ZP80) Galactokinase , complete | SNaPshot | TGATGGGCTCATGACTTTT | G/124/F | C | GCGAACCTGTTGGACTTGAT | GTTCACATCAAAAGCAGCCA |
| GLIP139 | GLIP ITAP | TC101210 | FH893911 | Similar to UP|Q86BN8 (Q86BN8) CG10371-PB, isoform B, partial (10%) | CAPS | AseI | 400+130 | 530 | AAGGATCTTGGTGTTCGTGG | TATGAAGTCAACAGCACGGC |
| GLIP163 | GLIP ITAP | TC97554 | FH893909 | Similar to UP|Q9LF37 (Q9LF37) ClpB heat shock protein-like, partial (11%) | CAPS | MnII | 70+175 | 70+16+159 | CCTCTTGACCGTGACCAAAT | CTTCACCGGTCTAGCACCAT |
| GLIP169 | GLIP ITAP | BG645063 | FH893908 | Similar to UP|Q9FHH4 (Q9FHH4) Diacylglycerol kinase-like protein, partial (14%) | CAPS | SmII | 290+130 | 420 | CCTTTCAGTGTGAGGGCAAT | GCGACATCAGCTTCAACAAA |
| GLIP171a | GLIP ITAP | TC110248 | NA | Weakly similar to UP|Q9AWB3 (Q9AWB3) Nuclear cap-binding protein CBP80, partial (11%) | Length | NA | - | 460 | TGGGAAGAAAAGTGCTGCTT | GCCTCGTTAATTGGTCTTCG |
| GLIP171a | GLIP ITAP | TC110248 | NA | Weakly similar to UP|Q9AWB3 (Q9AWB3) Nuclear cap-binding protein CBP80, partial (11%) | Length | NA | 410 | 390 | TGGGAAGAAAAGTGCTGCTT | GCCTCGTTAATTGGTCTTCG |
| GLIP185 | GLIP ITAP | TC95063 | FH893913 | Homologue to UP|HEM3_PEA (Q43082) Porphobilinogen deaminase, chloroplast precursor (PBG) (Hydroxymethylbilane synthase) (HMBS) (Pre-uroporphyrinogen synthase) | CAPS | MnII | 500 | 170+330 | GGAAATGTGCAAACAAGGCT | CATGATTCAGCGAAGCAAGA |
| GLIP245 | GLIP ITAP | TC104078 | FH893915 | Similar to F10O3.11 protein - Arabidopsis thaliana {Arabidopsis thaliana;}, partial (93%) | CAPS | MseI | 190+60 | 250 | ATTGACTGCTGTGGTGGTGA | CTTGCCCAATGGAAAGAAAA |
| GLIP253 | GLIP ITAP | TC107232 | FH893937 | Homologue of [UP|FKB2_VICFA](http://www.pir.uniprot.org/cgi-bin/upEntry?id=FKB2_VICFA) (Q41649) FK506-binding protein 2 precursor (Peptidyl-prolyl cis-trans isomerase) (PPIase) | CAPS | HinfI | 130+170 | 300 | TTGGGAATGTGTCTTGGTGA | TTTTGTCGTTCACTCCCACA |
| GLIP263 | GLIP ITAP | TC112179 | FH893936 | Similar to UP|Q6NL07 (Q6NL07) At1g13820, *partial (53%)* | CAPS | HgaI | 120+100 | 220 | CACTGGTGCAGAACAAGGAA | CAGAGAAACCCCAACCAAGA |
| GLIP265 | GLIP ITAP | CA923069 | FH893934 | Similar to UP|Q67XR9 (Q67XR9) MRNA, complete cds, clone: RAFL25-28-O12 (At1g29120), partial (27%) | CAPS | AseI | 340 | 150+190 | ATGAGCAAAGAAAGGCCAAA | TACCAGCACGTGGTTGATGT |
| GLIP291 | GLIP ITAP | TC94436 | FH893933 | Homologue to [UP|Q8W1A0](http://www.pir.uniprot.org/cgi-bin/upEntry?id=Q8W1A0) (Q8W1A0) Cysteine synthase | CAPS | HpyCH4IV | 190+30 | 220 | TGCAAAGGGAATGAAAGGAG | TGGACCGGTGGTTTCATAAT |
| GLIP307 | GLIP ITAP | TC103542 | FH893931 | Weakly similar to GB|AAP37738.1|30725432|BT008379 At4g13350 {Arabidopsis thaliana;} , *partial (7%)* | SNaPshot | CCACCAAGTGGGAAGAAGTT | T/190/F | A | GCGAATTAGTGAACCTCCCA | CAAAGGAGGCCCAATTGTTA |
| GLIP337 | GLIP ITAP | TC109547 | FH893939 | Similar to [UP|Q75HQ2](http://www.pir.uniprot.org/cgi-bin/upEntry?id=Q75HQ2) (Q75HQ2) Hypothetical protein P0636F09.16 | CAPS | ApoI | 420 | 190+180+50 | TGCAATGATTTGGGCACTTA | CTTTTGGCCAAGTACCAGGA |
| GLIP427 | GLIP ITAP | TC105548 | FH893928 | Similar to UP|Q8VX08 (Q8VX08) Phytoene desaturase precursor, *partial (18%)* | CAPS | FokI | 330+640 | 970 | CGTGCTTCTCCTCGTCCTAC | TTGCCTCCAGCAATATAGGC |
| GLIP429a | GLIP ITAP | CA918197 | NA | Weakly similar to [UP|Q9LTN0](http://www.pir.uniprot.org/cgi-bin/upEntry?id=Q9LTN0) (Q9LTN0) Arabidopsis thaliana genomic DNA chromosome 3 P1 clone: MTC11 | Length | NA | - | 900 | CGTGGACAACAAATTCATGC | GCACTTACCGGTCAATGGAT |
| GLIP429b | GLIP ITAP | CA918197 | NA | Weakly similar to [UP|Q9LTN0](http://www.pir.uniprot.org/cgi-bin/upEntry?id=Q9LTN0) (Q9LTN0) Arabidopsis thaliana genomic DNA chromosome 3 P1 clone: MTC11 | Length | NA | 530 | 510 | CGTGGACAACAAATTCATGC | GCACTTACCGGTCAATGGAT |
| GLIP451 | GLIP ITAP | TC108310 | FH893927 | Similar to [UP|Q8W4S5](http://www.pir.uniprot.org/cgi-bin/upEntry?id=Q8W4S5) (Q8W4S5) AT5g63710/MBK5_19 | CAPS | BstUI | 290+320 | 270+290+50 | TTCTAGCAGGGCTCCTTTCA | AGCTGAAACATGGGCTCACT |
| GLIP621a | GLIP ITAP | TC100832 | FH893925 | Homologue to [UP|Q8S2V7](http://www.pir.uniprot.org/cgi-bin/upEntry?id=Q8S2V7) (Q8S2V7) Putative sodium-dependent bile acid symporter | CAPS | HpyCH4V | 130+10 | 140 | CATGCTGCTGCATTTGCTAT | AGGAACAGCTACAAGGGGGT |
| GLIP621b | GLIP ITAP | TC100832 | NA | Homologue to [UP|Q8S2V7](http://www.pir.uniprot.org/cgi-bin/upEntry?id=Q8S2V7) (Q8S2V7) Putative sodium-dependent bile acid symporter | Length | NA | 500 | - | CATGCTGCTGCATTTGCTAT | AGGAACAGCTACAAGGGGGT |
| GLIP651a | GLIP ITAP | TC96712 | FH893887 | Similar to UP|Q9LVE0 (Q9LVE0) Nitrate transporter | CAPS | HpyCH4IV | 680 | 660+20 | TTGTTGCAAGCAATGGAGAG | AAGAAGGCCAAGCAAGTTGA |
| GLIP651b | GLIP ITAP | TC96712 | NA | Similar to UP|Q9LVE0 (Q9LVE0) Nitrate transporter | CAPS | HpyCH4IV | 230+160 | 390 | TTGTTGCAAGCAATGGAGAG | AAGAAGGCCAAGCAAGTTGA |
| GLP | Mt cross-species | AW126224 | FH893888 | Germin-like protein | CAPS | Hpy188III | 210+180 | 390 | GACTCAACACACTTGGTATATCTTTGGCTC | TTATCCACTTGAAAGGCTTTGGTGAGAAC |
| HBP2 | Mt cross-species | AW126142 | FH893878 | CASGL hemoglobin II | CAPS | ApoI | 320+130+20 | 470 | TTTATGGTCTCCAAAAGTGCAAACTTTGT | ATATTTGAGATTGCTCCATCAGCTCA |
| HYPTE3 | Mt cross-species | AI974791 | FH893876 | Hypothetical protein | CAPS | ApoI | 390 | 230+160 | TCGTCTCATGGTGGAATCGTGATGGT | TTCCTCCTTTAAACAAGCAAATTGGA |
| JUNBP | Mt cross-species | AW208151 | FH893875 | Putative JUN kinase activation domain binding protein | CAPS | MnII | 200+150 | 350 | TCTTCGTCATCATCTTCGGCGATAGCACA | GGATGTGAATGATACCATCCCACGACA |
| LG004 | MLG ITAP | TC208087 | NA | Weakly similar to [*GB|AAM63799*](http://www.ncbi.nlm.nih.gov/entrez/query.fcgi?cmd=search&db=protein&doptcmdl=genpept&term=AAM63799) unknown {Arabidopsis thaliana;} | Length | NA | 110 | - | CGAGGAAACGTTGCAGGTGG | CGTGAAGGGTTTGGCCAAGGC |
| LG007 | MLG ITAP | TC230161 | FH893895 | Similar to [*UP|Q9FPT2*](http://www.pir.uniprot.org/cgi-bin/upEntry?id=Q9FPT2) (Q9FPT2) Ubiquitin-specific protease 8 (Fragment) | CAPS | HindIII | 300+130 | 430 | TTGACGTTGATCTTTACAGCCAGG | TGCAAGAAGAACCTCTTGGACCAG |
| LG013 | MLG ITAP | TC205974 | FH893902 | Homologue to UP|Q94KS2 (Q94KS2) TGF-beta receptor-interacting protein 1, partial (57%) | CAPS | HinfI | 230+240 | 470 | CTGAGGCATCCTGACCACCTCC | CAGTCAATGCAGTTACCATGTCACC |
| LG018 | MLG ITAP | BM308011 | FH893900 | Similar to [*PIR|T49132*](http://pir.georgetown.edu/cgi-bin/nbrfget?xref=1&id=T49132) hypothetical protein F26G5.110 - Arabidopsis thaliana | CAPS | DdeI | 300+270 | 570 | CCTTGTCCCACGAAACATCAAGC | TCAGAAGAGCATCCATGCTGGC |
| LG023 | MLG ITAP | TC228354 | FH893899 | Homologue to UP|BSL2_ARATH (Q9SJF0) Serine/threonine protein phosphatase BSL2 (BSU1-like protein 2) , partial (42%) | CAPS | SspI | 350 | 220+130 | TCGGGGACAGCACAGCTTGG | CGATGCCATGTCCAAATTCCG |
| LG025 | MLG ITAP | TC228354 | FH893897 | Homologue to UP|BSL2_ARATH (Q9SJF0) Serine/threonine protein phosphatase BSL2, partial (42%) | SNaPshot | GTGTAGATCATACATGTGCTATTTGATAAT | C/210/F | T | TTGCGACCAAATGCTAGAGGTCC | AGCAAAACGCTCAAACCCATCC |
| LG031 | MLG ITAP | AC152751 | FH893905 | Weakly similar to putative Aminotransferase {Oryza sativa} [Oryza sativa (japonica cultivar-group)], partial (51%) | CAPS | HincII | 470 | 320+150 | CCTTCATACGTTTCATAGGAAGGGTC | CCGTTTCTGCCATCAATTCCG |
| LG033 | MLG ITAP | TC228615 | FH893894 | Similar to [*UP|Q9LP50*](http://www.pir.uniprot.org/cgi-bin/upEntry?id=Q9LP50) (Q9LP50) F28N24.10 protein | CAPS | HgaI | 480 | 300+180 | GCTGATATGAGGCGCGAAAGTG | GCCCCGCAGAGGGCAGAGG |
| LG034a | MLG ITAP | TC228615 | NA | Similar to [*UP|Q9LP50*](http://www.pir.uniprot.org/cgi-bin/upEntry?id=Q9LP50) (Q9LP50) F28N24.10 protein | Length | NA | - | 700 | TGCCCTCTGCGGGGCAGG | TTCTCCTCAGCCTCCCGTCG |
| LG034b | MLG ITAP | TC228615 | NA | similar to [*UP|Q9LP50*](http://www.pir.uniprot.org/cgi-bin/upEntry?id=Q9LP50) (Q9LP50) F28N24.10 protein | Length | NA | 270 | - | TGCCCTCTGCGGGGCAGG | TTCTCCTCAGCCTCCCGTCG |
| LG034c | MLG ITAP | TC228615 | NA | similar to [*UP|Q9LP50*](http://www.pir.uniprot.org/cgi-bin/upEntry?id=Q9LP50) (Q9LP50) F28N24.10 protein | Length | NA | 200 | - | TGCCCTCTGCGGGGCAGG | TTCTCCTCAGCCTCCCGTCG |
| LG036 | MLG ITAP | TC228227 | FH893893 | Similar to GB|AAL69531.1|18491125|AY074833 At2g47490/T30B22.21 {Arabidopsis thaliana;} , partial (61%) | CAPS | XcmI | 500 | 370+130 | GCATCCACGTTGACATATCCTCATG | GATTGGTGGCACAGCCTCGG |
| LG038 | MLG ITAP | AC152751 | FH893891 | Similar to UP|FUM1_ARATH (P93033) Fumarate hydratase 1, mitochondrial precursor(Fumarase 1) , partial (40%) | CAPS | NlaIII | 280+160 | 440 | GTTGAAACTAGTGGAGCCCTTAACAC | CCTCACACTGGGTCGGGTTAACC |
| LG041 | MLG ITAP | TC229193 | FH893890 | Similar to UP|FUM1_ARATH (P93033) Fumarate hydratase 1, mitochondrial precursor (Fumarase 1) , partial (40%) | CAPS | HphI | 240 | 170+70 | TGCTTGGAGATTCGTCTGCTTCC | CAACTGTTGCTGCCTTGTCATAACC |
| LG054 | MLG ITAP | AC147775 | FH893922 | Similar to UP|Q8H0P8 (Q8H0P8) RNA-bindinG protein, partial (60%) | CAPS | FokI | 220 | 130+90 | TCTGCTTATGACCGCTACCTTCAG | AATTGACATTCCGTCCATTTCGTG |
| LG068 | MLG ITAP | TC226707 | FH893892 | Similar to UP|Q7XA50 (Q7XA50) Sorbitol-like transporter, partial (62%) | CAPS | AciI | 100+90 | 190 | CGCACAGGATTTGGTGCAGTTCC | TCCTGGATTCATTTGGAAGACATTG |
| LG083 | MLG ITAP | AC138131 | FH893889 | Similar to GP|15010782|Gb T9J23.7/T9J23.7 {Arabidopsis thaliana}, partial (47%) | SNaPshot | GTAGAAAAATAAGGGAGAGCACATT | A/40/F | T | GGAGAACTGGCTCGGTATGCTGC | TGGGAATGTTGTGATGCTTCAACC |
| LG085 | MLG ITAP | AC138131 | FH893901 | Similar to TR:O82258 O82258 hypothetical 28.0 KDA protein | CAPS | HpyCH4V | 260 | 230+30 | GTGCCAGTTCCACCATCATAGCC | CAGCTCCAATCCAGCATCTTGC |
| LG088 | MLG ITAP | TC217560 | FH893881 | Similar to [*UP|Q6I5Y1*](http://www.pir.uniprot.org/cgi-bin/upEntry?id=Q6I5Y1) (Q6I5Y1) Hypothetical protein OJ1562_H01.4 | Length | NA | 500 | 511 | GCTCATGGAGGCAATGATGCTG | CATTGCCGCGACACAGAGGAC |
| LG093 | MLG ITAP | AC140032 | FH893874 | Similar to GB|AAM10290.1|20147147|AY091691 AT3G13930/MDC16_5, partial (74%) | CAPS | TaqI/TaqaI | 600 | 500+100 | GCATTGGATTATACCGACATTCCTG | GCTTCTTGCAATGAATTGAGTTGGG |
| LG101 | MLG ITAP | AC125473 | FH893896 | NA | SNaPshot | TTGAAATTACACAAGCTCAAA | C/177/R | T | GTGAGTCATACCAGCCTTGTAACCC | GAAGCGTGCTGCTCGTCACAG |
| LG102 | MLG ITAP | CO979090 | FH893898 | Unknown protein [Arabidopsis thaliana]. | SNaPshot | AACTCGTGCAAAATTTAATATAAAAA | A/330/F | C | GCCACACCTTGGTTCTAGAGGGC | GGCGTTTGAAGGAGCGTAAAGC |
| LG107 | MLG ITAP | TC229445 | FH893903 | Weakly similar to UP|Q9LI28 (Q9LI28) ESTs D23839(R0339), partial (19%) | SNaPshot | GTTGTTGCTGCATCCCTATAAA | T/300/F | A | CAGGCCTTCCTCCTCACATATCTG | GGTACTTGTGATAATTGGTTGGCTCC |
| LSSR31 | MLG ITAP | BG154045 | NA | Helicase, C-terminal [Medicago truncatula]. | Length | NA | - | 400 | TCCGAGGAGTTTGTGAAACC | TGCAAGCCCATAGCATCAA |
| LSSR5 | MLG ITAP | CA409926 | NA | PPD2 (PEAPOD 2) [Arabidopsis thaliana]. | Length | NA | 220 | - | GAAAGGTGAATGTGTATGATGGAGT | GGAGGATATTCCAGCATCTTATCT |
| LSSR6a | MLG ITAP | CA410040 | NA | Xyloglucan endotransglycosylase/hydrolase precursor XTH-3 [Populus tremula x Populus tremuloides]. | Length | NA | 580 | - | GTTGTTTTGGGACAACACCC | AAAACCCGAACCTGTGTAGC |
| LSSR9b | MLG ITAP | CA410979 | FH893877 | Putative lysosomal acid lipase [Arabidopsis thaliana]. | CAPS | Hpy8I | 170+220 | 390 | CAAAGGATGGTTTCTTGTTAGGTC | CCTCGCACATTTCCTACCCA |
| Lup011 | ML ITAP | BG154029 | FH893880 | Cytosolic malate dehydrogenase | CAPS | SspI | 50 | 100 | ctgtaatggttggtggattc | attccttcaggatcaatgc |
| Lup036 | ML ITAP | CA410134 | FH893917 | ATPDR1/PDR1 (PLEIOTROPIC DRUG RESISTANCE 1); ATPase | CAPS | MboII | 200 | 180 | ctgatatccattcacctcaag | aacacctggaagtccaacta |
| Lup052 | ML ITAP | BG149132 | FH893920 | Predicted protein [Physcomitrella patens subsp. patens]. | CAPS | MnII | 680 | 500+180 | ctggttctgttcttcaactgta | gtcatatcctttccatgcac |
| Lup066 | ML ITAP | CA409635 | FH893932 | Alpha-tubulin | SNaPshot | AGAAATTAGTTCAATTCCAATTGTC | A/100/R | T | gctatctatgacatttgcagg | attctaggataagggaccaagt |
| Lup091 | ML ITAP | CA410772 | FH893926 | Transmembrane protein | CAPS | ApoI | 300 | 210+90 | tctttaaaggtgtagctctggt | aggttggagtcagtcttagaaa |
| Lup099 | ML ITAP | CA410980 | FH893929 | Actin | CAPS | HinfI | 450 | 400+50 | agactttcaacgtacctgctat | accaagtactccgttaaatcac |
| Lup108 | ML ITAP | CA411183 | FH893923 | 40S ribosomal protein S12. | CAPS | XhoI | 200 | 100+100 | cagaacaacattgtaggcttc | tgtgcaagattgattctgag |
| Lup123 | ML ITAP | CA411479 | FH893935 | Cytosolic glutamine synthetase | CAPS | MboII | 90+60 | 150 | gctctcagatcttatcaacctt | acttcactatcttctccaggg |
| Lup172 | ML ITAP | BG154039 | NA | GTP-binding Dynamin-like protein | Length | NA | 280 |  | attatcttagctatctctcccg | catcaacagcattagttcct |
| Lup181 | ML ITAP | CA411421 | NA | Probable aquaporin TIP-type (MtAQP1). | Length | NA | - | 830 | cttcttggctccatcgtt | atagttcccaaactacccttct |
| Lup185 | ML ITAP | CA411496 | FH893883 | 40S ribosomal protein S3 | CAPS | AciI | 390+ | 630 | gaaggagaattagggagcttac | ccactttccatgacaaatct |
| Lup210 | ML ITAP | CA409635 | NA | Αlpha-tubulin | Length | NA | 480 | 370 | gcgctctcttgacattga | tgaagtggattctaggataagg |
| Lup242 | ML ITAP | CA410847 | NA | Glucose-6-phosphate/phosphate-translocator precursor | Length | NA | 660 | 510 | ttgtctctggtaattctcacac | agacatgtatgacacctgattg |
| Lup243 | ML ITAP | CA410908 | NA | Alpha-tubulin | Length | NA | 2kb | 1.8kb | ccgatcaacttactgatgaac | taatcatgtcctgcaactca |
| Lup246 | ML ITAP | CA411057 | NA | Eukaryotic initiation factor | Length | NA | 320 |  | actaatgcagatgagaggtctt | atccttgtcagagagagttcc |
| Lup266a | ML ITAP | CA411452 | NA | Cytochrome c | Length | NA | - | 800 | ggatattcctattcgacagcta | tatgcaataagatcagcacg |
| Lup266b | ML ITAP | CA411452 | NA | Cytochrome c | Length | NA | 560 | 540 | ggatattcctattcgacagcta | tatgcaataagatcagcacg |
| Lup266c | ML ITAP | CA411452 | FH893938 | Cytochrome c | CAPS | ApoI | 310 | 160+150 | ggatattcctattcgacagcta | tatgcaataagatcagcacg |
| Lup268 | ML ITAP | CA411479 | FH893930 | Glutamine synthetase cytosolic isozyme (Glutamate--ammonia ligase). | CAPS | HinfI | 300+50 | 350 | gctctcagatcttatcaacctt | tcacttcactatcttctccagg |
| Lup280 | ML ITAP | CA410338 | NA | 40S ribosomal protein S6 | Length | NA | 1.2 | 1.1 | ggatgtgacaaacaaggatt | gaacagactttctcctacgttct |
| Lup299 | ML ITAP | CA410686 | FH893886 | Ascorbate peroxidase | CAPS | ApoI | 240+160 | 400 | taagctatgggaaagtcgtacc | gcgatatcaagaccgttattag |
| Lup328 | ML ITAP | CA411108 | FH893910 | 40S ribosomal protein S23 (S12). | CAPS | NlaIII | 220+80 | 300 | ggcagacaagtcatacaagaa | aagcagcaatcttcttacca |
| Lup337 | ML ITAP | CA411368 | FH893914 | Similar to 40S ribosomal protein S14 | CAPS | AseI | 600 | 300+300 | aagaaactgttacacttggtcc | atagcagcataaggagatgact |
| Lup346 | ML ITAP | CA526341 | NA | Alpha-tubulin [Gossypium hirsutum]. | Length | NA | 840 | 870 | gtcaagccggtattcaagt | ccagatctacaaatatagcacg |
| MMK1a | Mt cross-species | AI974644 | FH893859 | Mitogen-activated protein kinase homolog | SNaPshot | GTTGTATATCATGTTTTTGATGGG | A/240/F | G | GATGGGGATCGAGAATATTCCGGC | CTTGTTGTCAAATGCATTTGCAATCTTC |
| MMK1b | Mt cross-species | AI974644 | NA | Mitogen-activated protein kinase homolog | Length | NA | - | 220 | GATGGGGATCGAGAATATTCCGGC | CTTGTTGTCAAATGCATTTGCAATCTTC |
| Ms/U182 | Mt cross-species | AJ410102 | NA | Proline-rich protein APG isolog | Length | NA | - | 420 | TACTGGGAGGTTTTGCAATGGCA | CAAATAAAGTCCTTGCAGCAGGTAG |
| Ms/U515 | Mt cross-species | AJ410128 | FH893862 | Chloroplast glyceraldehyde-3-phosphate dehydrogenase subunit B | SNaPshot | TCATGGGAGATGATATGGTTAA | G/450/F | A | GTTAAGGGAACCATGACAACCACA | CATTCATTGTCATACCAAGCAACCA |
| Ms/U121 | Mt cross-species | AW127371 | FH893840 | Ferredoxin NADP reductase precursor | CAPS | NdeI | 1.3 | 750+550 | TCCAACTCCACTTCTCTTCCGATCA | CAGTTTTGGAGTCTCCAAAATCACCA |
| NRT2 | Mt cross-species | AW225622 | NA | Putative high affinity nitrate transporter | Length | NA | 370 | 330 | GGAAGCTCCATGCATGGAGTA | ATTGCACCCATTGCAAGCCTTGAGA |
| PEPCASE | Mt cross-species | AW208231 | FH893861 | Phosphoenolpyruvate carboxylase | CAPS | MboII | 390 | 290+100 | TGGCAGCTATATAAGGCTCAGGAG | GAAATACTCAACAAAACGTGGTTCCTTGA |
| PESR1 | Mt cross-species | AA660526 | FH893837 | Pectinesterase | CAPS | PsiI | 260 | 190+70 | ACAAGCCTAGAAAATCCATTC | CGATATGAGTGTACTCAATTTGTGGTC |
| PGDH | Mt cross-species | AW126358 | FH893830 | Phosphogluconate dehydrogenase | SNaPshot | TTGGGTGAACTCGCCCG | A/238/R | T | GAGTTGAAGCTGCAAAGGTCTTTAAATCA | TGTATGAGCACCGAAGTAGTCTCGTTGA |
| PNDKN1 | Mt cross-species | AW127113 | FH893828 | Nucleoside diphosphate kinase I | CAPS | AseI | 950 | 610+340 | GGCCGAACAAACTTTCATCATGATCA | CCAGGCTCGGATTGAGCAGGGTTTGT |
| PPH | Mt cross-species | AI737531 | FH893860 | (p)ppGpp3-pyrophosphohydrolase | CAPS | HinfI | 170+110 | 280 | ATGTTCCTTGCCATGGCAGATGCAAGA | CGCCCAGAAATGACATGATAAGAAATG |
| PRAT | Mt cross-species | AW126346 | FH893870 | Phosphoribosyl anthranilate transferase | CAPS | Hpy8I | 350 | 200+150 | AGGAGGTAGTTGAGTACATGTCTGACA | GGAAGGAAATGTGTCAAATTCCTCATCA |
| PTDT | MLG ITAP | AI974616 | FH893872 | Homologue to UP|Q8MFR8 (Q8MFR8) Plastidic ATP/ADP transporter (Fragment), partial (51%) | CAPS | HpaI | 270 | 110+160 | GCAACTGGAATTGCTACATTCATAATGA | CGAATAAACTGTACTTAGCACTCTTGC |
| PTSB | Mt cross-species | AW127108 | NA | Proteasome beta subunit | Length | NA | 860 | 840 | ACTAAACAACACGCTAATTGGTCTCCA | ATGCCTAGCAGACAAAACCTTCTGCA |
| PTSS4 | Mt cross-species | AW225596 | FH893851 | Putative 26S Protease Subunit 4 | CAPS | Hpy188III | 260+130 | 390 | TGGGATTTTGTCTTTTGTTGATAAGGATCAG | TTGGACCATCTCCCAAGTACTTTTGTA |
| PUT3 | MLG ITAP | AW225627 | FH893849 | Similar to [*PIR|T01272*](http://pir.georgetown.edu/cgi-bin/nbrfget?xref=1&id=T01272) hypothetical protein At2g19240 [imported] - Arabidopsis thaliana | CAPS | TaqI | 330+510 | 840 | GAGTCGCTTTTTCCGCAATGCAGA | CTTAGTAATACAATGGACTGTATCTCA |
| RBPC/O | Mt cross-species | AW127446 | FH893865 | Ribulose-1,5-bisphosphate carboxylase/oxygenase activase precursor | CAPS | RsaI | 270 | 180+90 | CCCCATCATGATGAGTGCTGGAGA | TTGAGAAATCGTTACCGGTGACAATGATG |
| REP | Mt cross-species | AA660953 | FH893867 | Poly(A)+ RNA export protein | CAPS | TaqI | 200+180 | 380 | GCCAATTTCCTCGTCGCTACTTC | GCTAAAAGATTCATCTCCGGTATCCA |
| RNAR | Mt cross-species | AI974492 | FH893845 | Ribonucleotide reductase | CAPS | RsaI | 410+50 | 460 | GTTTGGCAGATTGTTGGGGTGAAGA | GGTAGGGCAATTGATGCAAGGTTACACA |
| SARB | Mt cross-species | AW127520 | FH893846 | Homologue to UP|SARA_ARATH (O04834) GTP-binding protein SAR1A | CAPS | MnII | 130+60+60 | 90+60+60+40 | GTTCATCTTTGATTGGTTCTATGGCATTC | ACCACCCAAATCAAAAGCTTTAAACTTGA |
| SAT | Mt cross-species | AW126397 | FH893858 | Sulfateadenylyl transferase | CAPS | AciI | 2x120+180 | 120+180 | GTATCATGATGGACTTGATCATTTTCGTC | AGCCTTTGCATGCCACTGCACCTCA |
| SHMT | Mt cross-species | AW127633 | NA | Serinehydroxymethyl transferase, mitochondrial precursor | Length | NA | 760 | 740 | ACCACAACTCACAAGTCACTTC | TTGCTGAGAACCTGCTCTTGGTATG |
| SUSY | Mt cross-species | AW126351 | FH893833 | Sucrose synthase (sucrose UDP glucosyl transferase) | CAPS | HpyCH4IV | 540 | 320+220 | TCGCAATGAACCACACAGATTTCA | GTCCAACCTTGCCATGGTGAAGATA |
| TBB2 | Mt cross-species | AW191276 | FH893852 | Tubulin beta-2 chain | CAPS | Tsp509I | 290 | 240+50 | TGTGGGATTCCAAGAACATGATGTG | TTCATACTCATCCTCCTCTGCAGTA |
| tRALS | Mt cross-species | AW126282 | FH893855 | Cytosolic tRNA-Ala synthetase | CAPS | XhoI | 380 | 290+90 | GGTCTGCGAGCTGTTTTTGGAGAAG | GCAATTCCCTCCTCAGCTAAAAGTG |
| UNK28 | Mt cross-species | AW126295 | FH893885 | Similar to UP|O80644 (O80644) Expressed protein, partial (65%) | CAPS | HpaII | 530 | 320+210 | GTAGATTCAATTCAAGTGCGAAAGGCTTTC | TACCAACCCATCAATGTTCTTCTAACTTTG |

NA: not applicable
